# Supplementary material for: Polyketide synthase gene clusters in the ericoid mycorrhizal fungus Oidiodendron maius: genome-wide prediction, expression profiling, and functional characterization of knockout mutants
Source: Front Plant Sci. 2026 Sep 16;17:1924430. doi: 10.3389/fpls.2026.1924430 (PMC13623903; doi:10.3389/fpls.2026.1924430)
Supplement: Supplementary file 1 [file SupplementaryFile1.zip › Supplementary Materials/Supplementary Folder 1.zip!/Oidma1_AssemblyScaffolds_Repeatmasked/knownclusterblast/cluster55/jgi.p_Oidma1_173667_mibig_hits.html]

| MIBiG Protein | Description | MIBiG Cluster | MiBiG Product | % ID | % Coverage | BLAST Score | E-value |
| --- | --- | --- | --- | --- | --- | --- | --- |
| AGO86665.1 | equisetin\_N-methyltransferase | BGC0001255 | Equisetin\_biosynthetic\_gene\_cluster | 35 | 89.218328841 | 198 | 1.8e-50 |
| AAD32742.2 | MmcR | BGC0000915 | Mitomycin\_biosynthetic\_gene\_cluster | 30 | 69.2722371968 | 75 | 1.7e-13 |
| AAZ78330.1 | OxyF | BGC0000254 | Oxytetracycline\_biosynthetic\_gene\_cluster | 24 | 90.8355795148 | 72 | 8.6e-13 |
| ACN64843.1 | PokMT2 | BGC0001061 | Polyketomycin\_biosynthetic\_gene\_cluster | 28 | 49.5956873315 | 59 | 7.5e-09 |
| BAB69170.1 | O-methyltransferase | BGC0000271 | Spore\_pigment\_biosynthetic\_gene\_cluster | 21 | 79.5148247978 | 56 | 1.1e-07 |
